# Naptha a Cure for Cholera—the Elixir of Voroniej

**Published:** 1848-08

**Authors:** 


ROYAL MEDICO-BOTANICAL SOCIETY OF LONDON.

*Naphtha a cure for Cholera—The Elixir of Voroniej.*—Mr. Guthrie, prior to reading certain communications which he had received from Circassia respecting the successful treatment of cholera in the Russian army in the Caucasus, observed that the authenticity of the documents which he was about to bring before the Society was undoubted, and that, as hitherto the profession had not entertained any definite views respecting cholera and its treatment, he considered it was highly satisfactory that a remedy had been discovered which exerted a specific action in this disease. This remedy was a singular one ; it was naphtha exhibited in small doses of from ten to twenty drops, the dose being repeated if required, which was rarely the case. The naphtha that was used was not the naphtha of the shops—not the petroleum, or Barbadoes tar, but a pure white, or rose-coloured naphtha, which was employed without being subject to distillation. It was in all probability the mineral naphtha which was obtained from Bakù, on the borders of the Caspian Sea. In order, however, to determine precisely the character and properties of this mineral, he (Mr. Guthrie) had sent to Circassia to procure some of it, and, as soon as it arrived, it should be placed in the hands of the secretary of the Society.

Mr. Guthrie then read extracts from letters from Dr. Andreyeoski, and from Prince Woronzow, the Russian Commander-in-Chief in Circassia. Dr. Andreyeoski says—"Naphtha, or petroleum, is not distilled, and the white is to be preferred; is an infallible remedy against the diarrhœa cholerica which prevails during certain seasons; and is given in the dose of from four to eight drops in a little brandy, white wine, or mint tea, taken cold. A single dose usually suffices to arrest the complaint. The evacuations, which in this species of diarrhœa are always liquid and glairy, become more solid, and less frequent. Sometimes the dose requires repetition at the end of two or three days. The diet should not be too strictly, but carefully regulated. In completely developed cholera of a deadly nature, from fifteen to twenty drops are to be given for a dose. If they are vomited the dose should be repeated; but a second is rarely required if the first be retained. It acts evidently on the skin and on the kidneys, and removes the cramps." In the first letter from the Prince Woronzow, dated Tiflis, March 1st, 1848, it is stated that "it is indisputable that most cholera cases begin with diarrhœa, and consequently, it is most important to act immediately and energetically against the first symptoms. The experience of the last year had proved, beyond a doubt, that naphtha was the best and easiest remedy in diarrhœa, whether it be simple diarrhœa, or the first symptoms of ensuing cholera. Dr. Andreyeoski thinks that the diarrhœa which precedes cholera is always without pain, and it is then that naphtha should immediately be resorted to; but in diarrhœa with pain in the bowels, he always employs opium. He first met the cholera last year at Tamikhan, where it prevailed to a very serious degree; the hospital he visited contained, the first day, more than 200 patients; the cases generally were very bad, and the mortality was great. On inquiry of the colonel commanding the Cossacks why there were so few Cossacks among the sick, he told them that he had made light of cholera, because they employed the elixir of Voroniej, which proved successful in almost every case. Andreyeoski immediately procured the recipe for the elixir, and on the first appearance of cholera in the convoy which accompanied the prince to the camp, he tried the drops with constant success. On examining the prescription, he found it to be a singular mixture of different matters, looking very like a quack medicine, but containing, among other strange, and, he thought, useless substances, some specific acting favorably in cholera, and he said that naphtha, one of the principal ingredients, might possibly be that specific. The stock of the elixir being soon exhaused, Dr. Andreyeoski determined to try naphtha alone, and, as he expected, it succeeded even in serious cases, but in mere diarrhœa the success was immediate. He had always, however, resorted to the elixir in cases to which he was apparently called too late—in the blue stage, accompanied by cramps, &c.,—but even in many of these advanced cases, naphtha alone had proved successful. Several cases had occurred among the officers, who had been quite blue, and suffered severely, but they were quite cured by it. As to simple diarrhœa during the existence of cholera, there was not a single case in which naphtha did not effect a cure when resorted to immediately. One of the Circassian chiefs was suddenly seized with cholera before Dr. Andreyeoski could see him; he had been bled, and was in the last stage of the disease. He was ordered some wine, and had two doses of the elixir, which, with friction and warm clothing, restored him to health, though his convalescence was tedious. The naphtha must be genuine white, or rose-colored, not black, nor brown, nor distilled, as that would be much too powerful." The following is an extract from a letter from Prince Woronzow, April 18th, 1848:—"In sending you the promised prescription for Dr. Andreyeoski's elixir, I must add that he recommends frictions of every part of the body during a real attack of cholera, besides the use of the elixir, and warm baths also, to alleviate the cramps. It must be remembered, that in almost all cases the real symptoms of cholera are preceded by diarrhœa without pain, to check which the naphtha drops have been, beyond comparison, the most successful remedy. If cholera appear abruptly, Dr. Andreyeoski advises the immediate employment of the elixir. If this be not within reach, then resort to the naphtha drops, as well as the warm baths, and especially to vigorous friction, to restore the circulation. Dr. Andreyeoski deprecates all bleeding, and mercurial medicines. If diarrhœa with pain occur even in cholera times, Dr. Andreyeoski treats it simply with opium, not considering it premonitory of cholera." The following is the formula for the elixir of Voroniej:—Rectified spirit of wine, seven ounces and a half; muriate of ammonia, a drachm; purified nitre, a drachm and twenty-five grains; pepper, a drachm and twenty-five grains; aqua regia, half a drachm; wine vinegar, a pint and a half; naphtha, half a drachm; olive oil, half an ounce; oil of peppermint, seven ounces. Digest for twelve hours, and strain. Take two teaspoonfuls for a dose every quarter of an hour.—*Lancet.*

